# Supplementary material for: Alpha-synuclein knockout impairs melanoma development and alters DNA damage repair in the TG3 mouse model in a sex-dependent manner
Source: Front Oncol. 2025 Mar 20;15:1554059. doi: 10.3389/fonc.2025.1554059 (PMC11967197; doi:10.3389/fonc.2025.1554059)
Supplement: Supplementary file 1 [file DataSheet1.docx]

***
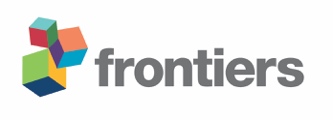
Supplementary Material***

**Alpha-synuclein knockout impairs melanoma development and alters DNA damage repair in the TG3 mouse model in a sex-dependent manner**

Moriah R. Arnold *et al.*

*Corresponding author: Vivek K. Unni (unni@ohsu.edu)

**
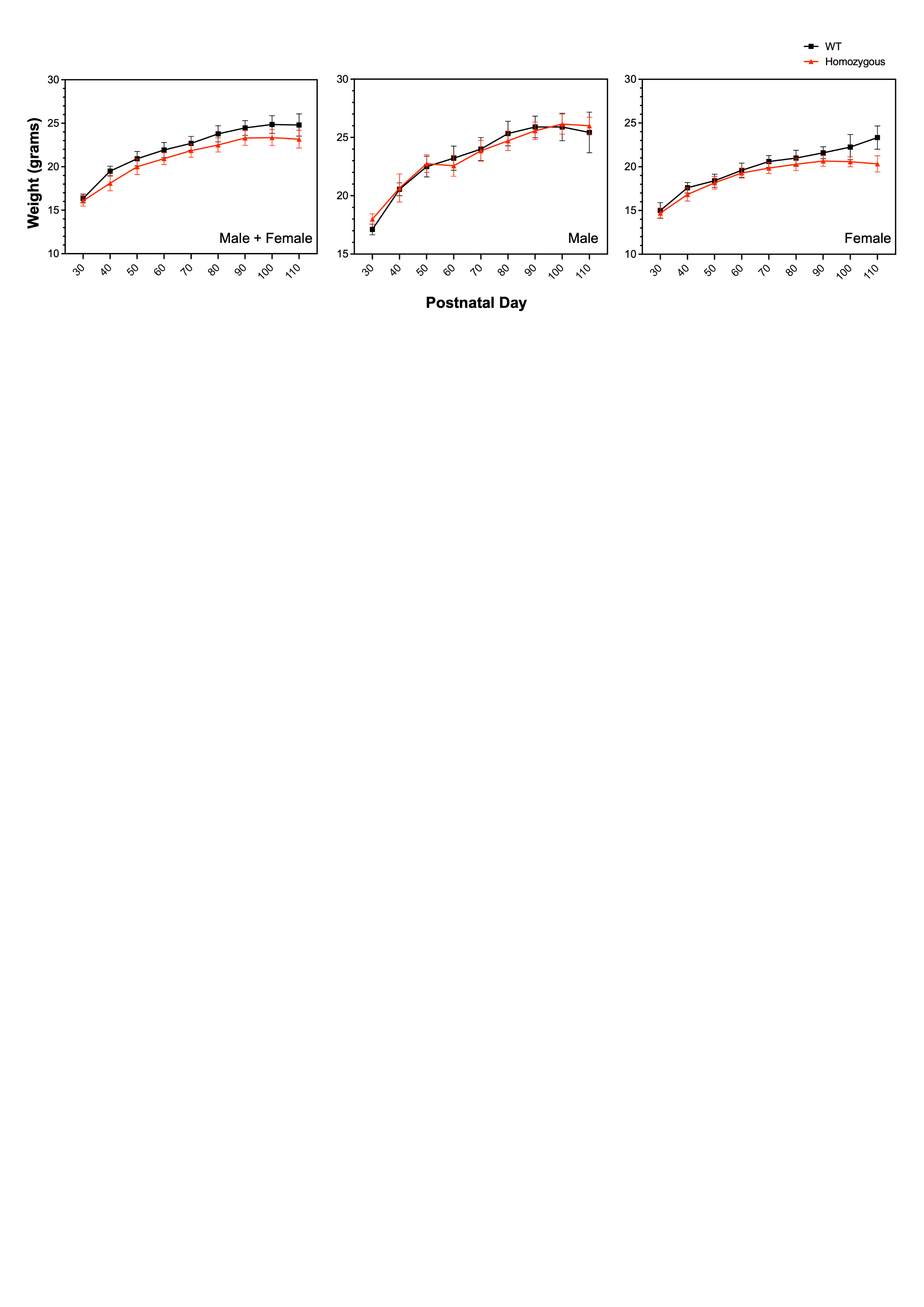
**

**Figure S1. Alpha-synuclein knockout does not affect mouse weight.**

Weight of TG3+/+*Snca*+/+ (n=15) and TG3+/+*Snca-*/- (n=14) from P30 to P110 (endpoint). Analysis was further stratified by sex with TG3+/+*Snca*+/+ male (n=10), TG3+/+*Snca*+/+ female (n=5), TG3+/+*Snca*-/- male (n=7), and TG3+/+*Snca*-/- female (n=7). Error bars represent Standard Error of the Mean (SEM). Statistical testing by two-way ANOVA. Weight is represented in grams.

**
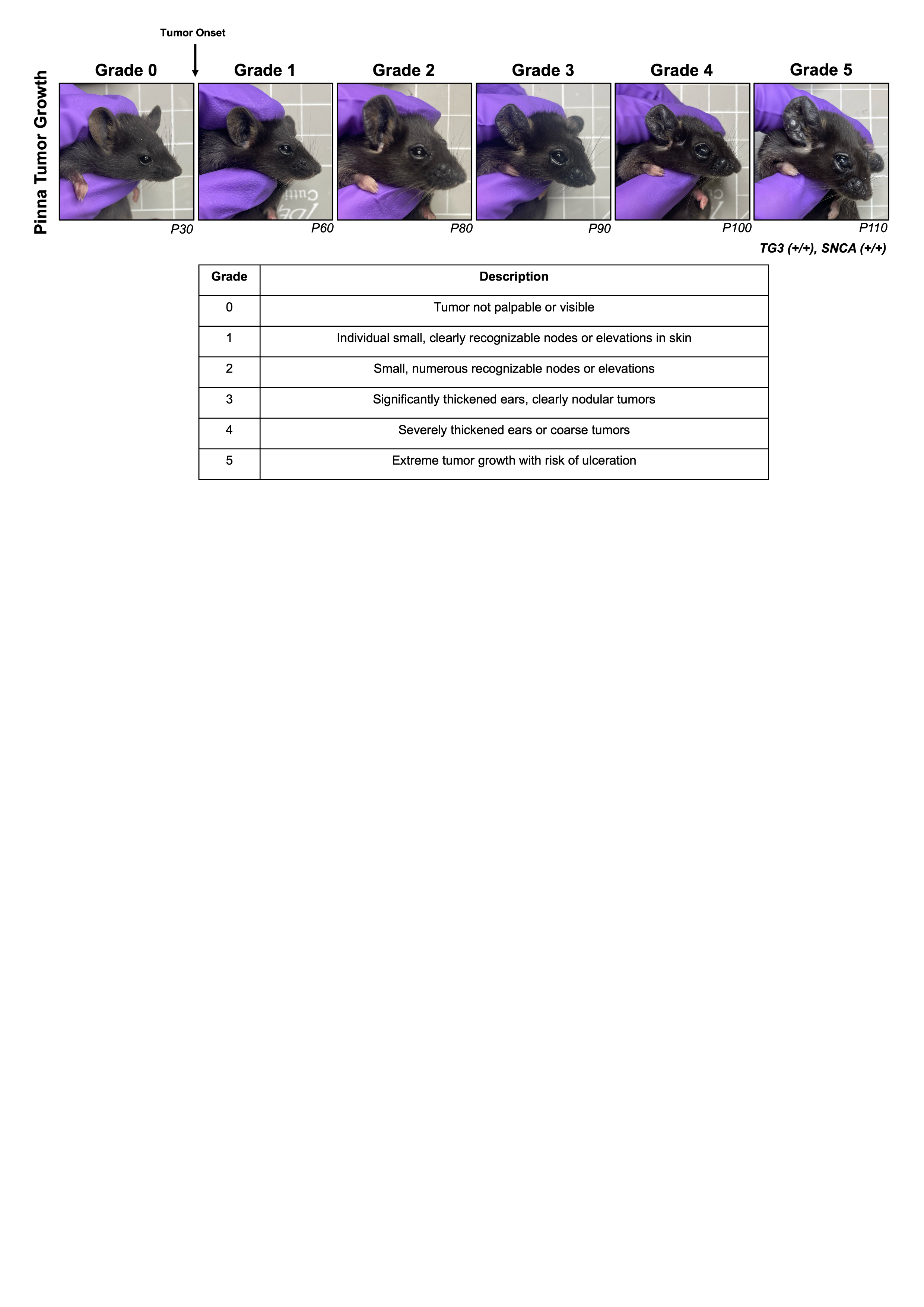
**

**Figure S2. Representative images and description of pinna tumor grading scale.**

| **Primer** | **Forward (5'-3')** | **Reverse (5'-3')** |
| --- | --- | --- |
| β-Actin | TGGAATCCTGTGGCATCCATGAAAC | TAAAACGCAGCTCAGTAACAGTCCG |
| Grm1 | GGGCAGGGAACGCCAATTCT | TGGAAGGGCTGCTGGGAGGG |
| Caspase-3 | AGCAGCTTTGTGTGTGTGATTCTAA | AGTTTCGGCTTTCCAGTCAGAC |
| Caspase-9 | TCCTGGTACATCGAGACCTTG | AAGTCCCTTTCGCAGAAACAG |
| RIP3 | AAGTGCAGATTGGGAACTACAACTC | AGAATGTTGTGAGCTTCAGGAAGTG |
| LC3B | CCCCACCAAGATCCCAGT | CGCTCATGTTCACGTGGT |
| Cdkn2a (p16) | CCCAACGCCCCGAACT | GCAGAAGAGCTGCTACGTGAA |

**Table S1. Primers used in qRT-PCRs.**
